# Upregulation of brain-derived neurotrophic factor in advanced gastric cancer contributes to bone metastatic osteolysis by inducing long pentraxin 3

**DOI:** 10.18632/oncotarget.10747

**Published:** 2016-07-21

**Authors:** Bongkun Choi, Eun-Jin Lee, Min-Kyung Shin, Young Soo Park, Min-Hee Ryu, Sang-Min Kim, Eun-Young Kim, Hyung Keun Lee, Eun-Ju Chang

**Affiliations:** ^1^ Department of Biomedical Sciences, Asan Medical Center, University of Ulsan College of Medicine, Seoul, Korea; ^2^ Department of Pathology, Asan Medical Center, University of Ulsan College of Medicine, Seoul, Korea; ^3^ Department of Oncology, Asan Medical Center, University of Ulsan College of Medicine, Seoul, Korea; ^4^ Department of Ophthalmology and Corneal Dystrophy Research Institute, Yonsei University College of Medicine, Seoul, Korea; ^5^ Cell Dysfunction Research Center, University of Ulsan College of Medicine, Seoul, Korea

**Keywords:** BDNF, PTX3, osteoblast, gastric cancer, bone metastasis

## Abstract

The brain-derived neurotrophic factor (BDNF) activates its receptor, tropomyosin receptor kinase B (TrkB; also called NTRK2) that has been shown to promote the malignant progression of several cancers. In this study, we investigated the clinical and biological significance of the BDNF/TrkB axis in the progression of human gastric cancer. The increased co-expression of the BDNF/TrkB axis was significantly correlated with bone metastatic properties in advanced gastric cancers. BDNF acting via TrkB receptors increased the levels of long pentraxin 3 (PTX3) that was related to bone metastatic status of gastric cancer by enhancing gastric cancer–osteoblastic niche interactions. In bone metastatic gastric cancer, PTX3 knockdown using small interfering RNA significantly inhibited BDNF-induced interactions of cancer cells with osteoblasts. Moreover, BDNF-derived PTX3 induction supported subsequent osteoclastogenesis, and this effect was significantly reversed by PTX3 silencing. These findings suggest that a functional interaction between BDNF/TrkB and PTX3 enhances the osteolysis of bone metastatic gastric cancer, thereby providing potential prognostic factors for the development of bone metastasis of gastric cancer.

## INTRODUCTION

As it advances, gastric cancer disseminates through the bloodstream and spreads to distant organs. Patients with advanced-stage gastric cancer frequently present with metastases in the peritoneum, lymph nodes, liver, lungs, and occasionally, bones [1]. The characteristic features of gastric cancer cell dissemination to the bone milieu include osteolytic bone destruction, which is associated with severe pain and pathological fractures [2, 3]. However, the mechanism by which gastric cancer enhances bone metastasis and the related prognostic factors remain largely unknown.

The establishment of a foothold within the bone marrow (BM) milieu is a prerequisite for bone metastatic cancer cells to parasitize the bone microenvironment and promote long-term cancer cell survival and subsequent metastatic growth [4, 5] by competing with hematopoietic stem cells (HSCs) for occupancy of the osteoblastic niche [6, 7]. In this setting, interactions of bone metastatic cancer cells with the BM environment stimulate osteoclast (OC) formation and activation, leading to osteolytic bone destruction, and in turn support tumor growth in BM, thereby establishing a “vicious cycle” [4, 5]. Therefore, elucidation of the complex interactions between bone metastatic gastric cancer cells and the BM milieu could provide novel treatment strategies to suppress gastric cancer–osteoblastic niche interactions and thus improve patient outcomes.

Brain-derived neurotrophic factor (BDNF) activates the high-affinity tropomyosin receptor kinase B (TrkB; also called NTRK2) [8]. Several studies have implicated BDNF and TrkB in the pathogenesis of various human malignancies, including not only neuronal tumors such as neuroblastoma [9] but also non-neuronal tumors such as lung cancer, hepatocellular carcinoma, ovarian cancer, breast cancer, pancreatic ductal carcinoma, prostate cancer, and multiple myeloma [10–14]. In addition, elevated expression of the BDNF/TrkB axis has been found to significantly correlate with disease progression and poor prognosis in gastric cancer in humans [15, 16]. BDNF-induced TrkB activation confers antiapoptotic, tumorigenic, invasive, and metastatic capacities upon cancer cells [17–20]. On the other hand, BDNF has been reported to stimulate receptor activator of nuclear factor kappa B ligand (RANKL) production from osteoblasts (OBs), thereby contributing to the formation of osteolytic bone lesions from multiple myeloma [21, 22]. Because RANKL is known to be an essential osteogenic factor that directly induces osteoclastogenesis [23], BDNF is considered an osteoclastogenic factor in bone metastatic cancer. However, no as-yet defined mechanism links the BDNF/TrkB pathway with cancer metastasis to a bone niche and subsequent osteolysis.

Here we identified elevated expression of the BDNF/TrkB axis in human advanced gastric cancers with bone metastatic properties. BDNF stimulates long pentraxin 3 (PTX3) expression through TrkB, thereby promoting interactions between bone metastatic gastric cancer cells and OBs and subsequent osteoclastogenesis. These findings provide evidence of an association of BDNF/TrkB with PTX3 in osteolytic bone metastases of gastric cancer as well as a better understanding of the mechanisms by which the BDNF/TrkB axis exerts its effects on gastric cancer biology.

## RESULTS

### Expression of the BDNF/TrkB axis is elevated in advanced human gastric cancers with bone metastatic potential

**Figure 1:**
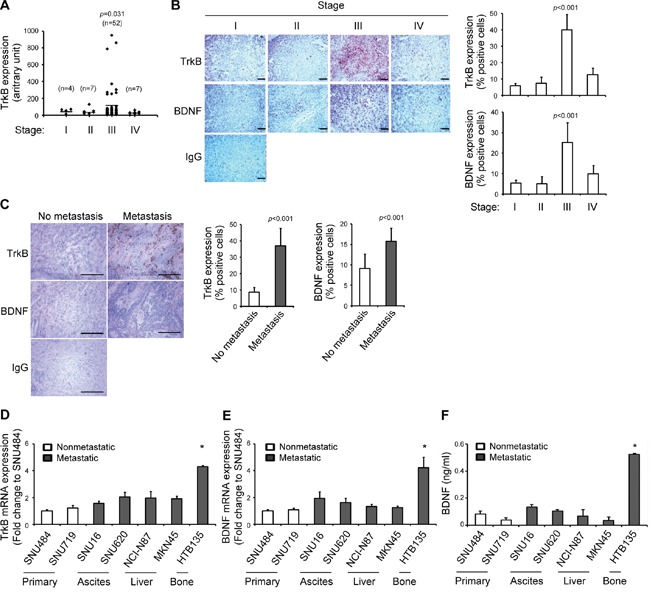
Elevated tropomyosin receptor kinase B (TrkB) and brain-derived neurotrophic factor (BDNF) expression levels in tumors from patients with advanced gastric cancer and bone metastatic gastric cancer cell lines **A.** Elevated TrkB transcript expression in patients with stage III gastric cancer. TrkB mRNA expression was analyzed in gastric tumor tissues from patients with sequential-staged gastric cancer: I (n=4), II (n=7), III (n=52), and IV (n=7). Expression data were extracted from the Gene Expression Omnibus (GEO) dataset GSE27342 and used to compare the TrkB expression status. Bars indicate the mean values of samples in each group (*P*=0.031 in comparison with stage I). *P* values were obtained using the Wilcoxon rank-sum test. **B.** Elevated TrkB and BDNF protein expression in patients with stage III gastric cancer. IHC was used to evaluate the expression levels of TrkB and BDNF proteins in gastric tumor biopsy tissues obtained from patients with sequentially staged gastric cancer (stages I–IV). IgG indicates rabbit control antibodies. Scale bar: 200 μm. TrkB and BDNF expression levels are represented as percentages of immunoreactive cells in gastric cancer tissue specimens. Data are shown as the means ± standard deviations (SD; *P <0.05 in comparison with Stage I). **C.** IHC was used to evaluate the expression levels of TrkB and BDNF proteins in gastric tumor biopsy tissues obtained from patients with no metastasis (n=8) and metastasis (n=9). IgG indicates rabbit control antibodies. Scale bar: 200 μm. TrkB and BDNF expression levels are represented as percentages of immunoreactive cells in gastric cancer tissue specimens. Data are shown as the means ± standard deviations (SD; *P <0.05 in comparison with no metastasis). **D** and **E.** Increased TrkB and BDNF mRNA expression in the bone metastatic gastric cancer cell line HTB135. TrkB (D) and BDNF (E) mRNA expression levels were determined by quantitative real-time polymerase chain reaction (qRT-PCR) in human nonmetastatic (SNU-484, SNU-719) and metastatic (SNU-16, SNU-620, NCI-N87, MKN45, and HTB135) gastric cancer cell lines. The expression levels are presented relative to those of glyceraldehyde 3-phosphate dehydrogenase (GAPDH), which was used as a reference gene. Ascites: peritoneal cavity fluid. Data are shown as means ± SD (*P <0.05 in comparison with SNU484 cells). **F.** Elevated BDNF protein expression in HTB135 cells. BDNF protein expression levels in culture media from human gastric cancer cell lines were determined using an enzyme-linked immunosorbent assay (ELISA). Data are shown as means ± SD (*P <0.05 in comparison with SNU484 cells). *P* values were obtained using Student's *t* test.

Patients with advanced-stage gastric cancer (stage III–IV) exhibited a much higher frequency of metastasis to distant organs [24] and more frequently died of recurrence within 2 years after gastrectomy [25]. To investigate the association between the TrkB gene expression signature and gastric cancer development, we analyzed a public cohort of 70 patients with early-stage (stage I–II) and late-stage (stage III–IV) malignant gastric cancer from the Gene Expression Omnibus (GEO, GSE27342) [26]. TrkB mRNA expression was significantly higher in patients with stage III gastric cancer than in those with stage I–II or IV gastric cancer (Figure 1A, *P*=0.031). In light of our finding that stage III gastric tumors express higher levels of TrkB mRNA, we next investigated through an immunohistochemistry (IHC) analysis whether the elevated protein levels of TrkB and BDNF would correlate with disease severity in patients with gastric cancer. Clinical specimens were obtained from patients with gastric cancers ranging from stage I to IV (Supplementary Table S1). IHC analysis revealed that advanced gastric tumors from stage III patients exhibited more robust TrkB immunoreactivity than tumors from in stage I–II or IV patients (Figure 1B, *P* <0.001). BDNF expression was also increased in gastric tumors from stage III patients in comparison with those from other-stage patients (Figure 1B, *P* <0.001). Moreover, gastric tumors with metastases showed increased expression levels of both TrkB and BDNF when compared to those without metastases (Figure 1C, *P* <0.001).

To determine the potential involvement of elevated TrkB expression in gastric cancer metastasis to distant organs, we compared TrkB mRNA expression levels between nonmetastatic (SNU-484 and SNU-719) and metastatic (SNU-16, SNU-620, NCI-N87, MKN45, and HTB135) gastric cancer cell lines. TrkB transcripts were detectable only at low levels in nonmetastatic gastric cancer cells (Supplementary Figure S1). In contrast, modest but insignificant increased TrkB mRNA expression was detected in the SNU-620 (ascites; peritoneal cavity fluid), NCI-N87 (liver), and MKN45 (liver) metastatic cancer cell lines (Figure 1D). Most notably, the HTB135 bone metastatic gastric cancer cell line [27] exhibited the highest TrkB mRNA level among all analyzed metastatic gastric cancer cell lines, as determined by real-time polymerase chain reaction (qRT-PCR) (Figure 1D, *P* <0.05).

Similarly, HTB135 bone metastatic cells expressed the highest level of BDNF mRNA among all gastric cancer cell lines (Figure 1E, *P* <0.05). Moreover, HTB135 cells secreted a higher mean amount of BDNF protein into conditioned media (CM) (0.531 ± 0.018 ng/ml) than nonmetastatic (0.060 ± 0.030 ng/ml), ascites (0.119 ± 0.021 ng/ml), or liver (0.049 ± 0.037 ng/ml) metastatic gastric cancer cells (Figure 1F, *P* <0.05), as measured by an enzyme-linked immunosorbent assay (ELISA); these results suggest that bone metastatic gastric cancer cells express elevated levels of BDNF/TrkB. HTB135 cells were selected among the studied cell lines for further *in vitro* analysis because these cells expressed relatively high levels of BDNF and TrkB. Taken together, these results suggest that elevated TrkB and BDNF expression in advanced gastric cancer correlates with bone metastatic properties.

### Long pentraxin 3 (PTX3) expression is associated with tumor severity in patients with gastric cancer with bone metastatic potential

**Figure 2:**
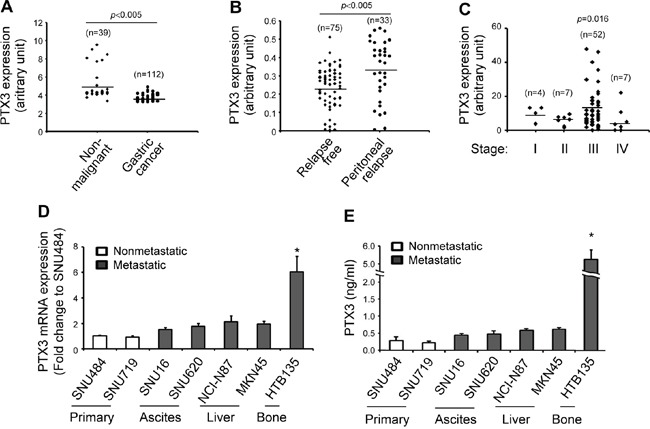
Comparison of long pentraxin3 (PTX3) expression levels in gastric cancer patients, advanced human gastric cancers, and bone metastatic gastric cancer cells **A.** Decreased PTX3 transcript expression in patients with gastric cancer. PTX3 gene expression was analyzed in a cohort of 151 human nonmalignant (n=39) and gastric cancer (n=112) specimens (GSE37023; *P* <0.005 in comparison with nonmalignant specimens). **B.** PTX3 mRNA expression was elevated in patients with peritoneal relapsed gastric cancer. PTX3 expression in human gastric tumors that did not (n = 75) or did relapse into the peritoneum (n=33) was analyzed using publicly available microarray data (GSE15081; *P* <0.005 in comparison with relapse-free gastric cancer). **C.** Increased expression of PTX3 mRNA in patients with stage III gastric cancer. PTX3 gene expression analysis in gastric tumor tissues from patients with sequential-staged gastric cancer was evaluated as described in Figure 1A (GSE27342; *P*=0.016 in comparison with stage I). *P* values were obtained using the Wilcoxon rank-sum test. **D.** Elevated PTX3 mRNA expression in the bone metastatic gastric cancer cell line HTB135. PTX3 mRNA expression levels were determined using qRT-PCR, as described in Figure 1D. **E.** Elevated PTX3 protein expression in HTB135 cells. PTX3 protein expression levels in culture media were determined as described in Figure 1F. Data are shown as means ± standard deviations (*P <0.05 in comparison with SNU484 cells). *P* values were obtained using Student's *t* test.

We next analyzed GEO (GSE27342 and GSE37023) [28] to identify potential genes upregulated by TrkB and observed a positive correlation of TrkB expression with PTX3 in patients with gastric cancer (Supplementary Figure S2A, correlation coefficient=0.468, *P* <0.0001). However, there was no significant correlation of BDNF expression with PTX3 expression (Supplementary Figure S2B, correlation coefficient=0.188, *P*=0.094). Elevated PTX3 expression has been associated with an increased risk of several malignancies, including glioma, liposarcoma, lung carcinoma, and pancreatic carcinoma [29–32]. In contrast, PTX3 expression is suppressed in other human tumors such as leiomyosarcoma and colorectal cancer [33]. This suggests a controversial role of PTX3 in the progression of malignancies in humans. To investigate the association between the PTX3 gene expression signature and gastric cancer development, we analyzed a public cohort of 151 nonmalignant (n=39) and gastric cancer (n=112) specimens (GSE37023) [28] and observed significantly lower levels of PTX3 in gastric cancer tissues than in nonmalignant tissues (Figure 2A, *P* <0.005). Next, to further support the association between PTX3 expression and metastatic potential in gastric cancer, PTX3 gene expression profiles from a cohort of 108 gastric cancer tissues associated or not associated with peritoneal relapse (GSE15081) were analyzed. Peritoneal relapse is one of the most common features of tumor progression associated with advanced gastric cancer [34]. Gene expression data from these patients with gastric cancer demonstrated that relapsed gastric cancer tissues expressed higher levels of PTX3 than relapse-free tissues (Figure 2B, *P* <0.005), indicating an increase in PTX3 expression in relapsed gastric cancer, despite a lower level of PTX3 expression in primary gastric cancer than in nonmalignant tissues. Using IHC, we previously reported a general increase in PTX3 expression in patients with advanced gastric cancer with relapse-correlated metastasis [35]. Consistent with this finding, an additional analysis of a cohort of 70 patients with early-stage (stage I–II) and late-stage (stage III–IV) malignant gastric cancer (GSE27342) [26] demonstrated significantly higher PTX3 expression in patients with stage III gastric cancer than in those with stage I–II or IV gastric cancer (Figure 2C, *P*=0.016). Collectively, these findings indicate that compared with nonmalignant specimens, PTX3 expression is repressed in patients with gastric cancer, although elevated PTX3 expression in gastric cancer may correlate with relapse-correlated metastasis. Similar to the pattern observed for BDNF/TrkB, PTX3 mRNA expression was distinctly elevated in bone metastatic HTB135 cells compared with other cells (Supplementary Figure S3 and Figure 2D). As shown in Figure 2E, the concentration of secreted PTX3 protein from HTB135 cells (5.62 ± 0.26 ng/ml, *P* <0.05) was much higher than that from other cells (0.44 ± 0.16 ng/ml), suggesting a relationship of the elevated expression levels of PTX3 and the BDNF/TrkB axis with the bone metastatic status in patients with advanced gastric cancer.

### BDNF induces PTX3 expression through TrkB signal that promotes chemotactic migration and binding of gastric cancer cells to OBs

**Figure 3:**
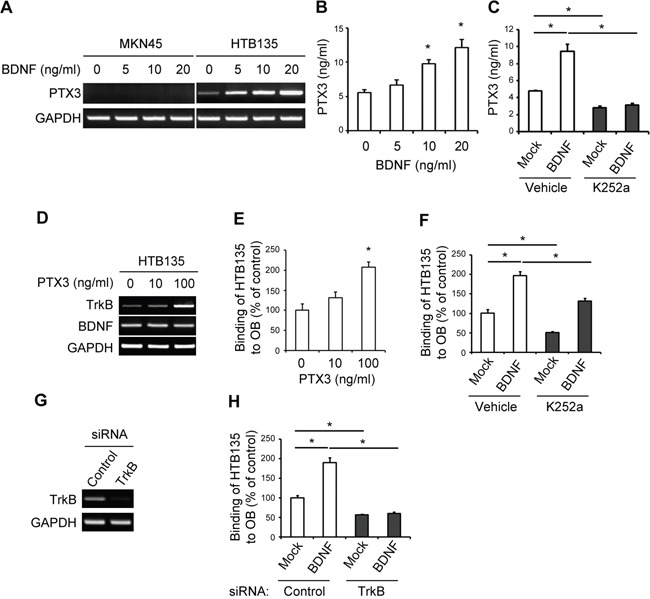
BDNF induces PTX3 expression and promotes bone metastatic gastric cancer cell binding to osteoblasts (OBs) via TrkB signaling **A.** BDNF induces a dose-dependent increase in PTX3 mRNA expression in HTB135 cells. MKN45 and HTB135 cells were treated with BDNF (0, 5, 10, and 20 ng/ml), and PTX3 mRNA expression levels were determined using RT-PCR. GAPDH was included as a control. **B.** BDNF triggers PTX3 protein secretion from HTB135 cells. PTX3 protein levels in conditioned media (CM) of HTB135 cells treated with increasing concentrations of BDNF for 48 h were evaluated with ELISA (*P <0.05 in comparison with vehicle control). **C.** BDNF-induced PTX3 upregulation is TrkB-dependent. HTB135 cells were pretreated with K252a (50 nM) and subsequently treated with BDNF (20 ng/ml). ELISA was performed to examine the concentration of PTX3 protein in CM. Data are shown as means ± standard deviations (SD; *P <0.05 in comparison with mock control). **D.** TrkB transcript levels increased upon PTX3 stimulation in HTB135 cells. HTB135 cells were treated with the indicated concentrations of PTX3. TrkB and BDNF mRNA expression levels were determined using RT-PCR. GAPDH was included as a control. **E.** PTX3 enhanced the binding of HTB135 cells to OBs. OBs were treated with different concentrations of PTX3 (0, 10, or 100 ng/ml), following which CellTracker™ Green 5-chloromethylfluorescein diacetate (CMFDA)-labeled HTB135 cells were layered onto OBs for 6 h. Thereafter, the ability of the HTB135 cells to bind to OBs following PTX3 treatment was evaluated using a fluorescent plate reader. Values represent the percentage of vehicle treatment (*P <0.05 in comparison with vehicle control). **F.** TrkB signal blockade attenuated the BDNF-induced increase in HTB135 cell binding to OBs. HTB135 cells were pretreated with K252a (50 nM) and subsequently treated with BDNF (20 ng/ml), following which the binding capacity of HTB135 cells for OBs was examined. Values represent percentages of the control. Data are shown as means ± SD (*P <0.05 in comparison with mock control). **G.** HTB135 cells were transfected with either negative control or TrkB specific siRNA and RT-PCR was performed to determine the level of TrkB mRNA. **H.** HTB135 cells were transfected with either negative control or TrkB targeting siRNA and treated with BDNF (20 ng/ml). The binding capacity of the HTB135 cells to osteoblasts was examined. Bars indicate the mean and standard deviations (SD) (*p < 0.05 in comparison with mock control). *P* values were obtained using Student's *t* test.

Given the upregulation of BDNF/TrkB and PTX3 expression observed in bone metastatic gastric cancers (Figures 1 and 2), we assumed that BDNF, a specific ligand of TrkB, induces PTX3 expression via TrkB signaling in bone metastatic gastric cancer cells. BDNF stimulation of bone metastatic HTB135 cells augmented PTX3 mRNA expression in a dose-dependent manner, whereas BDNF had no effect on PTX3 expression in liver metastatic MKN45 cells, as demonstrated by RT-PCR (Figure 3A). Consistent with these results, PTX3 protein expression was distinctly increased in HTB135 cells treated with 10 and 20 ng/ml BDNF, as determined by ELISA (Figure 3B, *P* <0.05). Several lines of evidence suggest that BDNF exerts its effects by interacting with a specific TrkB receptor [36, 37]. We therefore addressed whether TrkB activation may be involved in BDNF-induced PTX3 expression and found that K252a, a Trk pharmacological inhibitor, almost completely diminished the BDNF-induced increase in PTX3 protein expression, as shown by ELISA (Figure 3C, *P <0.05). These data suggest that TrkB is a functional receptor that promotes the effect of BDNF on PTX3 upregulation. Conversely, PTX3 at a concentration of 100 ng/ml markedly stimulated the transcriptional expression of TrkB mRNA but not BDNF mRNA in HTB135 cells (Figure 3D), implying the presence of reciprocal regulation between PTX3 and TrkB expression in bone metastasis of gastric cancer.

Bone-metastasized tumor cells compete with HSCs to occupy the osteoblastic niche during the process of bone-associated metastasis [6, 7, 34, 38]. We next hypothesized that elevated PTX3 expression in bone metastatic HTB135 cells stimulates the interaction of bone metastatic gastric cancer cells with OBs. Indeed, PTX3 promoted the migration of HTB135 cells toward OBs, as shown in a Transwell assay (Supplementary Figure S4, *P* <0.05) and further enhanced the binding of HTB135 cells to OBs (Figure 3E, *P* <0.05), emphasizing the distinct role of PTX3 in bone metastatic gastric cancer–osteoblastic niche interactions. Similar to PTX3, BDNF promoted bone metastatic gastric cancer–OB interactions (Figure 3F, white bars at left, *P* <0.05); however, the blockade of TrkB activation by either K252a (Figure 3F, gray bars at right, *P* <0.05) or TrkB specific small interfering RNA (siRNA) (Figure 3H, gray bars at right, *P* <0.05) hindered the BDNF-induced interactions of these cells, thereby indicating the requirement of TrkB activation in this interaction.

### BDNF-derived PTX3 induction stimulates binding of gastric cancer cells to OBs and supports OC formation

**Figure 4:**
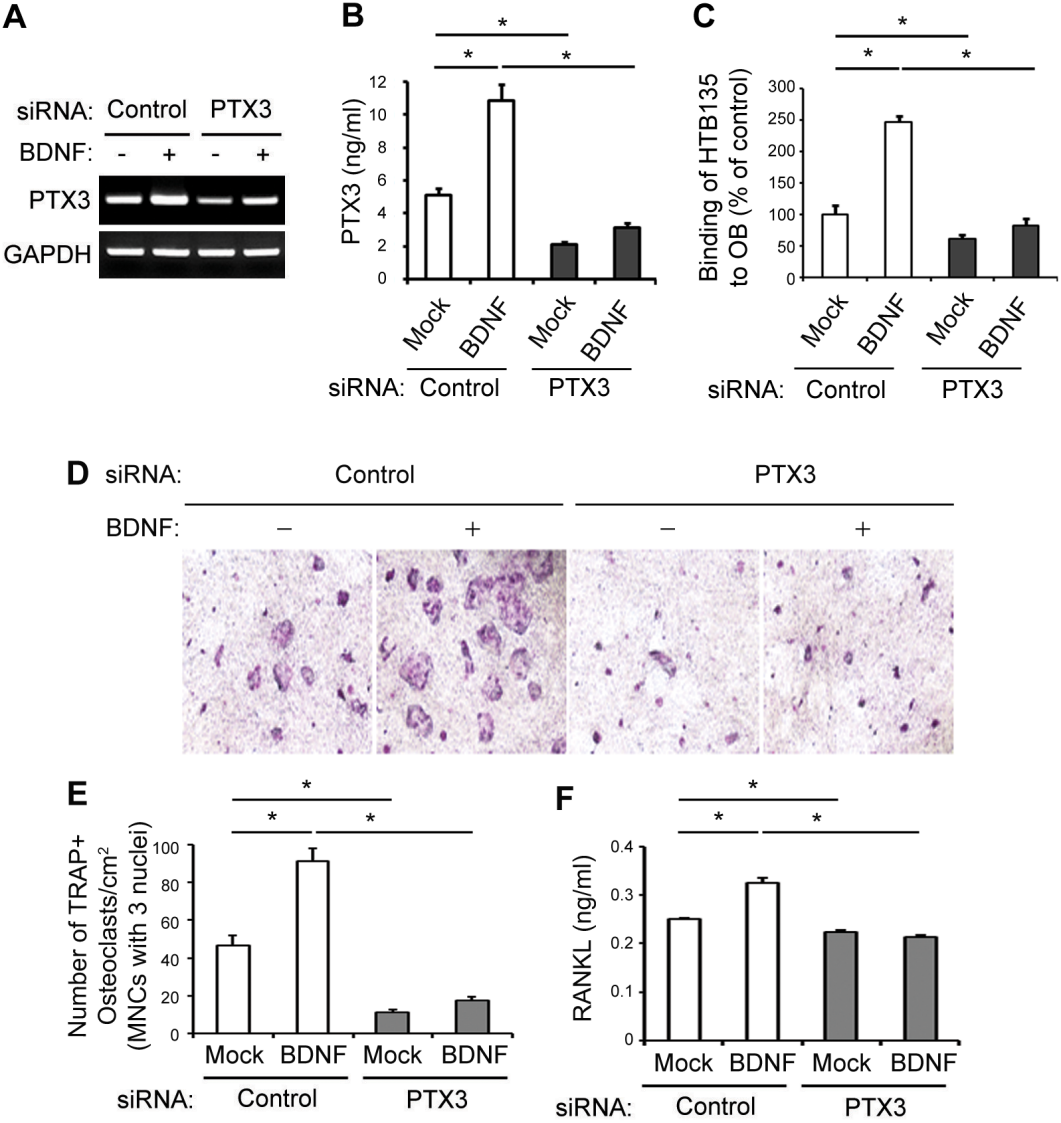
BDNF-derived PTX3 promotes bone metastatic gastric cancer cell binding to OBs and subsequent osteoclast (OC) formation **A.** PTX3 silencing reversed the BDNF-mediated induction of PTX3 mRNA expression. HTB135 cells were transfected with either PTX3-targeting or negative control siRNA and treated with BDNF (20 ng/ml) for 24 h. RT-PCR was performed to determine the level of PTX3 mRNA expression. **B.** PTX3 knockdown abrogated the BDNF-mediated induction of PTX3 protein expression. HTB135 cells were transfected with either PTX3-specific or negative control siRNA and treated with BDNF (20 ng/ml) for 48 h. PTX3 protein levels in CM were assayed by ELISA. **C.** The loss of PTX3 in HTB135 cells inhibited the BDNF-induced increase in interactions between HTB135 cells and OBs. HTB135 cells were transfected with either PTX3-targeting or negative control siRNA and treated with BDNF (20 ng/ml). The binding capacity of HTB135 cells for OBs was examined. Values represent percentages of the control (*P <0.05 in comparison with mock control). **D.** PTX3 silencing diminished BDNF-induced OC formation. Representative images of tartrate-resistant acid phosphatase (TRAP)-positive multinucleated cells (TRAP+MNCs) from a co-culture system are shown. HTB135 cells were transfected with either PTX3-targeting or negative control siRNA, treated with BDNF (20 ng/ml), and loaded in the upper chamber of a Transwell system. Murine bone marrow (BM) cells and OBs were co-cultured in the lower chamber. TRAP staining was performed to evaluate the formation of TRAP-positive OCs, and photographic images were obtained. **E.** The number of TRAP+MNCs counted from Figure 4D is depicted. The enhanced osteoclast formation induced by BDNF was reversed by PTX3 silencing (*P <0.05 in comparison with mock control). **F.** Loss of PTX3 reversed the BDNF-induced upregulation of receptor activator of nuclear factor kappa-B ligand (RANKL) expression. The concentration of soluble RANKL protein in the CM from Figure 4D was measured by ELISA. Data represent the means ± standard deviations (*P <0.05 in comparison with mock control). *P* values were obtained using Student's *t* test.

To verify the potential involvement of PTX3 in the BDNF-induced promotion of gastric cancer cell binding to OBs, we silenced PTX3 expression in HTB135 cells using PTX3-specific siRNA. PTX3-targeting siRNA successfully knocked down endogenous PTX3 expression in HTB135 cells, as shown by both RT-PCR (Figure 4A) and ELISA (Figure 4B, *P* <0.05). In HTB135 cells, PTX3 silencing completely obviated the BDNF-induced increase in HTB135 cell–OB interactions (Figure 4C, *P* <0.05), suggesting that BDNF-induced PTX3 is the predominant factor responsible for the interaction of bone metastatic gastric cancer cells with OBs.

A previous report documented the involvement of the BDNF/TrkB axis in the induction of OC formation and activation in the context of multiple myeloma, wherein RANKL is overexpressed by OBs, followed by BDNF stimulation [22]. We previously reported that bone metastatic breast cancer cell-derived PTX3 enhanced osteoclastogenesis by upregulating RANKL expression in OBs [39]. Therefore, we reasoned that BDNF-derived PTX3 induction in bone metastatic gastric cancer cells may upregulate RANKL expression in OBs, thereby stimulating OC formation. BDNF treatment led to a significant increase in OC formation, as demonstrated by an approximately twofold increase in tartrate-resistant acid phosphatase (TRAP)-positive multinucleated cells (TRAP+MNCs) in comparison with vehicle control-treated cells (Figure 4D and 4E, *P* <0.05). Importantly, these phenomena could be completely reversed by PTX3 silencing (Figure 4D and 4E, *P* <0.05), suggesting that BDNF supports OC formation by upregulating PTX3. The protein level of RANKL, a critical osteoclastogenic factor primarily produced by OBs, was elevated in CM from a co-culture of BM and pre-OBs in the presence of BDNF; however, PTX3 silencing completely abolished this BDNF-induced increase in RANKL expression in OBs (Figure 4F, *P* <0.05). Taken together, these results indicate that in addition to its positive effect on the interaction between bone metastatic gastric cancer cells and OBs, BDNF may play a functional role in enhancing osteoclastogenesis by upregulating PTX3, which in turn promotes RANKL production from OBs.

## DISCUSSION

Advanced-stage gastric cancer cells can acquire a migratory capacity and metastasize to other tissues [1, 40]. Previous reports have demonstrated significant correlations of elevated BDNF/TrkB axis expression with disease progression and poor prognosis in patients with gastric cancer [15, 16]. The present study is the first to support a strong possible relationship between elevated BDNF/TrkB expression and bone metastatic potential in advanced-stage gastric cancers (Figure 1). Several studies have defined osteoblastic lineage cells in BM as key components of the HSC niche [6, 7]. In BM, disseminated cancer cells from solid tumors compete with HSCs for occupancy of the BM niche on endosteal bone surfaces [38]. Previously, OB-produced RANKL was found to promote the migration capacities of cancer cells expressing the RANK receptor [41]. Similar to this RANKL–RANK axis, it is tempting to postulate that BDNF expressed by OBs [42, 43] promotes the migratory capacity of disseminated gastric cancer cells expressing elevated levels of TrkB, a specific receptor of BDNF.

The present study indicates that primary gastric cancers express lower levels of PTX3 than nonmalignant tissues; however, bone metastatic gastric cancer cells were found to express elevated levels of PTX3 (Figure 2). Although PTX3 is a representative marker of cancer-related inflammation [29], whether PTX3 acts as a tumor promoter or tumor suppressor remains controversial. Most other studies have suggested that increased PTX3 expression promotes the severity of tumor malignancy in various types of cancer, including liposarcoma, lung cancer, glioma, and pancreatic carcinoma [29–32]. In contrast, Bonavita et al. [33] recently reported the epigenetic repression of PTX3 via the promoter region in selected human cancers such as leiomyosarcoma and colorectal cancer, thereby proposing that PTX3 serves as an extrinsic oncosuppressor that restrains complement-dependent tumor-promoting inflammation. Given the associations of the PTX3 expression profile and its role in inflammation-mediated gastric cancer progression [35], it is reasonable to assume that PTX3 acts as a tumor promoter by enhancing inflammation rather than as a tumor suppressor, at least in metastatic advanced-stage gastric cancer.

PTX3 protein, which is induced by the BDNF/TrkB axis, facilitates the interaction of gastric cancer cells with OBs (Figure 3), thereby suggesting a potential role of PTX3 in establishing bone metastatic footholds of gastric cancer cells in BM. The findings that TrkB activation is an obligatory event in PTX3 expression and, in turn, that PTX3 stimulates TrkB expression (Figure 3) clearly support the idea that PTX3 is a possible mediator in the bone metastatic gastric cancer–OB interaction with respect to the BDNF/TrkB axis. However, PTX3 exerted no influence on BDNF expression (Figure 3), and no significant correlation was observed between PTX3 and BDNF expression levels in patients with gastric cancer (Supplementary Figure S2). Because enforced TrkB expression promotes the metastatic capacity of lung adenocarcinoma [44] and is sufficient to suppress apoptosis, tumor formation, and metastasis in an animal model [45, 46], it appears beneficial that PTX3-induced upregulation of TrkB rather than BDNF allows PTX3 to remain responsive to BDNF stimulation. Moreover, PTX3 induced bone metastatic gastric cancer cell proliferation through activation of Akt and inhibited apoptosis while PTX3 silencing reduced proliferation and increased apoptosis in HTB135 cells (Supplementary Figure S5).

The following steps occur once the cancer cells have invaded BM: first, BDNF derived from bone metastatic gastric cancer can stimulate TrkB on OBs [42, 43] to directly promote RANKL production [21, 22]. Second, TrkB activation by BDNF, produced by metastatic advanced gastric cancer cells, can induce PTX3 expression in the bone microenvironment, thereby contributing to an inflammatory osteoclastogenic condition by stimulating RANKL production from OBs. Here we found that BDNF promoted the secretion of PTX3 from gastric cancer cells in humans (Figure 3) and, in turn, PTX3 induced RANKL secretion from OBs to further amplify OC formation in a co-culture system (Figure 4), thereby mimicking the BM environment [47] and reinforcing the idea that BDNF and PTX3 likely contribute to the bone-destructive process and disease progression in gastric cancer. Our recent study provided further support for a role of PTX3 in osteoclastogenesis by demonstrating a correlation between elevated PTX3 in bone metastatic breast cancer and osteolytic function [39]. Taken together, these results suggest that BDNF is an important factor that contributes to the RANKL pool directly or via PTX3 upregulation in the bone, thereby suggesting that BDNF eventually creates a vicious cycle between OCs and bone metastatic gastric cancer cells to promote the osteolytic process and gastric cancer tumor growth.

**Figure 5:**
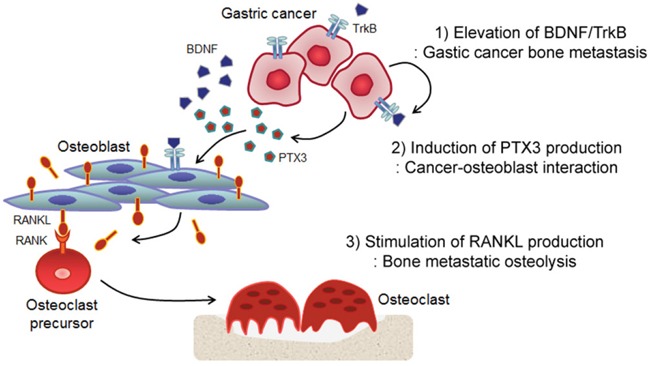
Hypothetical model of BDNF- and PTX3-induced osteolysis in bone metastatic gastric cancer **1.** BDNF/TrkB stimulates PTX3 gene expression. **2.** BDNF promotes the interaction between bone metastatic gastric cancer cells and OBs via PTX3 signaling. **3.** BDNF enhances RANKL production and secretion from OBs via PTX3 upregulation. **4.** Finally, PTX3-induced RANKL stimulates osteoclastogenesis.

In conclusion, we have demonstrated a functional interaction between BDNF/TrkB and PTX3 in advanced gastric cancer and have provided evidence that these proteins may enhance the interaction between bone metastatic gastric cancer cells and OBs, thereby leading to osteolysis (Figure 5). Our findings suggest that an assessment of the BDNF/TrkB and/or PTX3 expression status in gastric tumors allows clinicians to predict metastatic potential and improve the prognosis of patients with advanced gastric cancers and could further guide the development of better therapeutic regimens to improve outcomes in patients with gastric cancer.

## MATERIALS AND METHODS

### Cells and reagents

The HTB135 and NCI-N87 human gastric cancer cell lines were obtained from the American Type Culture Collection (ATCC, Manassas, VA, USA). The SNU series of gastric cancer cell lines (SNU-16, 484, 620, and 719) was obtained from the Korea Cell Line Bank (Seoul, Korea). HTB135 cells were maintained in Dulbecco's modified Eagle's medium (DMEM; Hyclone/Thermo Fisher Scientific Inc., Logan, UT, USA) supplemented with 10% fetal bovine serum, 50 units/ml streptomycin (Life Technologies, Carlsbad, CA, USA), and 50 μg/ml penicillin (Life Technologies). All other cell lines were cultured in RPMI-1640 (Hyclone/Thermo Fisher Scientific Inc.) supplemented with 10% FBS and were maintained at 37°C in a 5% CO_2_ incubator. Recombinant human PTX3 and BDNF proteins and rabbit anti-human PTX3, BDNF, and RANKL antibodies (Abs) used in ELISA analysis were purchased from R&D Systems (Minneapolis, MN, USA). Anti-BDNF and TrkB Abs for immunohistochemical analyses were purchased from Abcam (Cambridge, MA, USA). The EnVision™ G|2 Doublestain System, Rabbit/Mouse (DAB+/Permanent Red) was purchased from Dako (Carpinteria, CA, USA). K252a and a tartrate-resistant acid phosphatase (TRAP) kit were obtained from Sigma–Aldrich (St. Louis, MO, USA).

### IHC

Under protocol no. 2011-0484, approved by the Institutional Review Board (IRB) of Asan Medical Center (Seoul, Korea), gastric cancer tissue specimens were collected after surgical removal from patients with the Declaration of Helsinki. All patients provided written informed consent. Patient information is described in Supplementary Table S1. IHC was performed using the EnVision™ G|2 Doublestain System and Rabbit/Mouse (DAB+/Permanent Red), according to the manufacturer's protocol (Dako). Frozen tissues were cryosectioned in 5-μm-thick slices and dried for 5 min before fixation with 4% paraformaldehyde for 15 min. Sections were then permeabilized with 0.25% Triton X-100 in PBS for 10 min and blocked with a dual endogenous enzyme-blocking reagent for 30 min at room temperature before incubation with a primary antibody against TrkB or BDNF for 1 h at RT; subsequent detection was performed using the polymer/HRP System. Corresponding rabbit sera were used as negative controls. Following immunostaining, all slides were counterstained with Mayer's hematoxylin (Sigma–Aldrich).

### RT-PCR and qRT-PCR

Total RNA was isolated from cells using the RNeasy Mini Kit (QIAGEN, Hilden, Germany), according to the manufacturer's protocol. First-strand cDNA was synthesized from the total RNA of each sample using the RevertAid First Strand cDNA Synthesis Kit (Thermo Fisher Scientific Inc., Lafayette, CO, USA). The resulting cDNA was amplified using the DreamTaq Amplification Kit (Thermo Fisher Scientific Inc.). PCR was performed using a T100™ Thermal Cycler (Bio-Rad Laboratories, Hercules, CA, USA). The following primer sequences were used: human PTX3: 5′-CAT CTC CTT GCG ATT CTG TTT TG-3′ (sense) and 5′-CCA TTC CGA GTG CTC CTG A-3′ (antisense), human TrkB: 5′-GTT TCA TAA GAT CCG ACT GGA TGG-3′ (sense) and 5′-TGC TGC TTA GCT GCC TGA GAG TTA-3′ (antisense), human BDNF: 5′-AGC CTC CTC TTC TCT TTC TGC TGG A-3′ (sense) and 5′-TCC CGC CCG ACA TGT CCA CT-3′ (antisense), and human glyceraldehyde 3-phosphate dehydrogenase (GAPDH): 5′-AGC CAC ATC GCT CAG ACA-3′ (sense) and 5′-GCC CAA TAC GAC CAA ATC C-3′ (antisense). The amplified products were electrophoresed on 2.0% (w/v) agarose gels and visualized using ethidium bromide staining under ultraviolet light. qRT-PCR analysis was performed in optical 96-well plates with using *Power* SYBR Green *1-Step* Kit and ABI 7000 Real Time PCR System (Applied Biosystems, Foster City, CA, USA), according to the manufacturer's instructions. Gene expression was normalized to that of GAPDH, which was used as an internal control.

### ELISA

The concentrations of secreted BDNF, PTX3, or RANKL protein in conditioned media (CM) were determined using a BDNF-, PTX3-, or RANKL-specific sandwich ELISA system (R&D Systems), according to the manufacturer's protocol. Serial dilutions of recombinant BDNF, PTX3, or RANKL in culture media were used as standards. In brief, cell culture supernatants were added to the ELISA plates and washed with PBS containing 0.05% Tween 20. After incubation with biotinylated mouse anti-BDNF, PTX3, or RANKL Abs, horseradish peroxidase-conjugated streptavidin was added to the plates. Tetramethyl benzidine substrate was used to develop the colorimetric reaction, and the absorbance at OD 450 nm was determined using a microtiter plate reader (Bio-Rad Laboratories, Hercules, CA, USA). All samples in each experiment were examined at least in triplicate.

### siRNA transfection

The transfection of PTX3 or TrkB-targeting siRNAs was performed as described previously [47]. A combination of four selected siRNA oligonucleotide sequences, SMARTpoolsiRNA-targeting PTX3 or TrkB (ON-TARGET plus human PTX3 or TrkB), and control siRNA were obtained from Thermo Scientific Dharmacon. siRNAs were transfected into HTB135 cells using RNAiMAX (Life Technologies). Silencing efficiency was determined using RT-PCR or ELISA, as described above.

### OC formation

Primary mouse OBs and BM cells were isolated as described previously [47]. For the co-culture experiment, HTB135 cells transfected with negative control or PTX3-specific siRNA, followed by treatment with vehicle or BDNF (20 ng/ml), were loaded on the upper wells. Fresh BM cells (3 × 10^5^ per well) and calvarial pre-OBs (2 × 10^4^ per well) were co-cultured in the lower compartments of the Transwell System (Costar, Corning, NY, USA) in the presence of vitamin D3 (10^−8^ M) and prostaglandin E2 (10^−6^ M) for 7 days. The cells were stained for TRAP (Sigma–Aldrich) to detect OC formation.

### *In vitro* binding assay

Cell-to-cell binding assays were performed as described previously [38]. Primary mouse OB precursor cells were isolated from 1-day-old mouse calvariae. Murine calvarial OBs were plated onto 96-well plates at a concentration of 2 × 10^4^ cells/well in growth medium, and the cultures were incubated for 1 day. HTB135 cells (10^4^ cells) were labeled with CellTracker™ Green 5-chloromethyl fluorescein diacetate (CMFDA; Invitrogen) and layered onto OBs. Binding assays were performed in PBS. After extensive washing, any remaining fluorescence was quantified as a measure of HTB135 cell binding to OBs using a fluorescent plate reader (Spectra GEMINIXS; Molecular Devices, Sunnyvale, CA, USA).

### Analysis of PTX3 expression in gastric cancer using public microarray gene expression datasheets

A public cohort of 151 human nonmalignant (n=39) and gastric cancer (n=112) specimens was analyzed utilizing published data from GEO (GSE37023) to evaluate the PTX3 gene expression profile. A publicly available gastric cancer microarray datasheet (GSE27342) was also used to evaluate the TrkB, BDNF, and PTX3 gene expression profiles of 70 patients diagnosed with different stages of cancer (stages I–IV). A public microarray datasheet was downloaded from the GEO database (GSE15081) to compare PTX3 expression between nonrelapsed and relapsed gastric cancer cells (108 primary gastric cancer tissues). Observed differences with *P* values of <0.05 were considered statistically significant (Wilcoxon rank-sum test).

### Statistical analysis

TrkB, BDNF, and PTX3 gene expression profiles in patients with gastric cancer were analyzed using the Wilcoxon rank-sum test. All quantitative experiments were performed at least in triplicate, and data are expressed as the means ± standard deviations of a single representative experiment. Mean values between groups were compared, and statistical significance was determined using Student's *t* test unless otherwise indicated. A *P* value of <0.05 was considered statistically significant.

### Supplementary materials

Supplementary material is available at *Oncotarget*.

## SUPPLEMENTARY FIGURES AND TABLE


