# Supplementary material for: Upregulation of brain-derived neurotrophic factor in advanced gastric cancer contributes to bone metastatic osteolysis by inducing long pentraxin 3
Source: Oncotarget. 2016 Jul 21;7(34):55506–17. doi: 10.18632/oncotarget.10747 (PMC5342432; doi:10.18632/oncotarget.10747)
Supplement: Supplementary file 1 [file oncotarget-07-55506-s001.pdf]

Upregulation of brain-derived neurotrophic factor in advanced gastric cancer contributes to bone metastatic osteolysis by inducing long pentraxin 3

SUPPLEMENTARY FIGURES AND TABLE

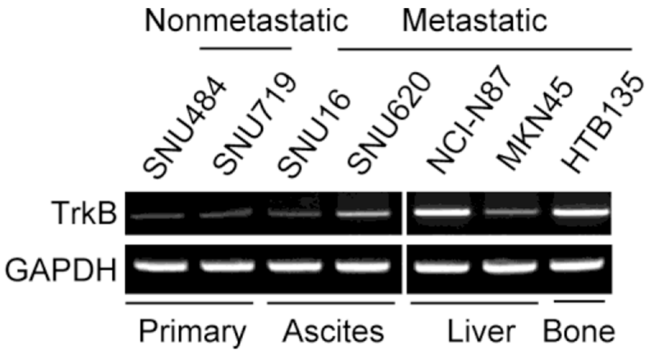

Supplementary Figure S1: Elevated tropomyosin receptor kinase B (TrkB) expression in bone metastatic gastric cancer cells. TrkB mRNA expression levels in human nonmetastatic (SNU-484 and SNU-719) and metastatic gastric cancer cell lines (SNU-16, SNU-620, NCI-N87, MKN45, and HTB135) were determined using RT-PCR. GAPDH was included as a control. Ascites: peritoneal cavity fluid.

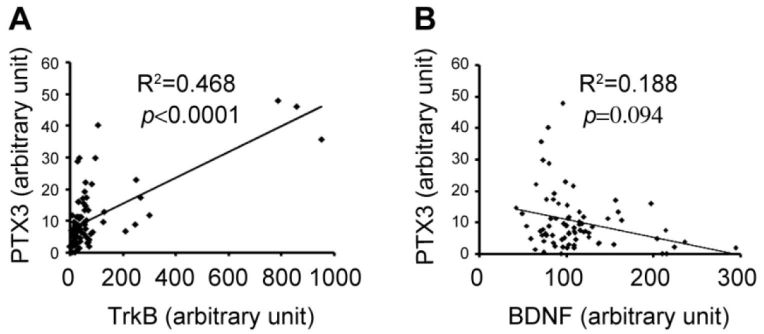

Supplementary Figure S2: Positive correlation between PTX3 and TrkB expression levels in human gastric cancer patients. A. The correlation between PTX3 and TrkB expressions was analyzed in human gastric cancer patients. Expression data were extracted from Gene Expression Omnibus (GEO) dataset GSE27342 (correlation coefficient=0.468, P <0.0001, n=70). **B**. The expression levels of PTX3 and brain-derived neurotrophic factor (BDNF) were analyzed in the same dataset (GSE27342, correlation coefficient=0.188, P =0.094, n=70). P values were analyzed using the Pearson correlation method and Prism software (GraphPad Software, San Diego, CA, USA).

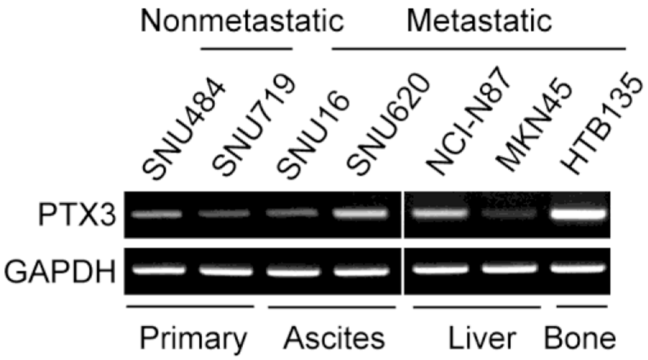

Supplementary Figure S3: Elevated PTX3 expression in bone metastatic gastric cancer cells. PTX3 mRNA expression levels in human nonmetastatic (SNU-484 and SNU-719) and metastatic gastric cancer cell lines (SNU-16, SNU-620, NCI-N87, MKN45, and HTB135) were determined using RT-PCR. GAPDH was included as a control. Ascites: peritoneal cavity fluid.

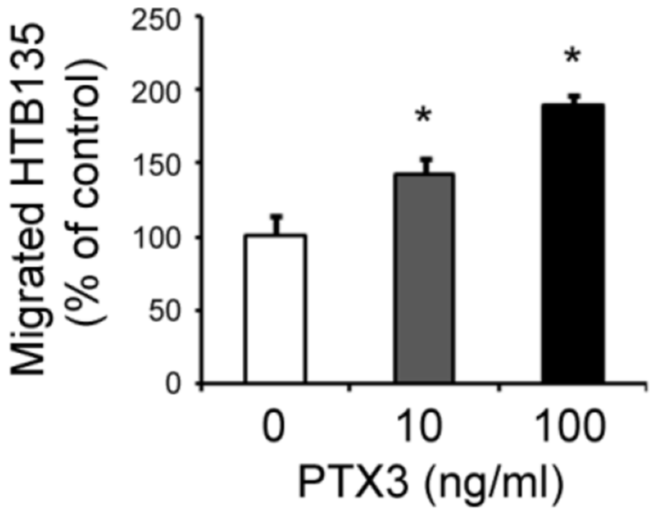

Supplementary Figure S4: Long pentraxin 3 (PTX3) enhances gastric cancer cell migration toward osteoblasts (OBs). OBs cultured in the lower compartment of the Transwell system were treated with the indicated concentrations of PTX3. CellTracker™ Green 5-chloromethyl fluorescein diacetate (CMFDA; Invitrogen)-labeled HTB135 cells (1.2×10^6) were added to the upper chamber of each Transwell and allowed to migrate for 6 hours, after which the number of HTB135 cells in the lower chamber was counted.(*P <0.05 in comparison with mock control). P values were obtained using Student's t test.

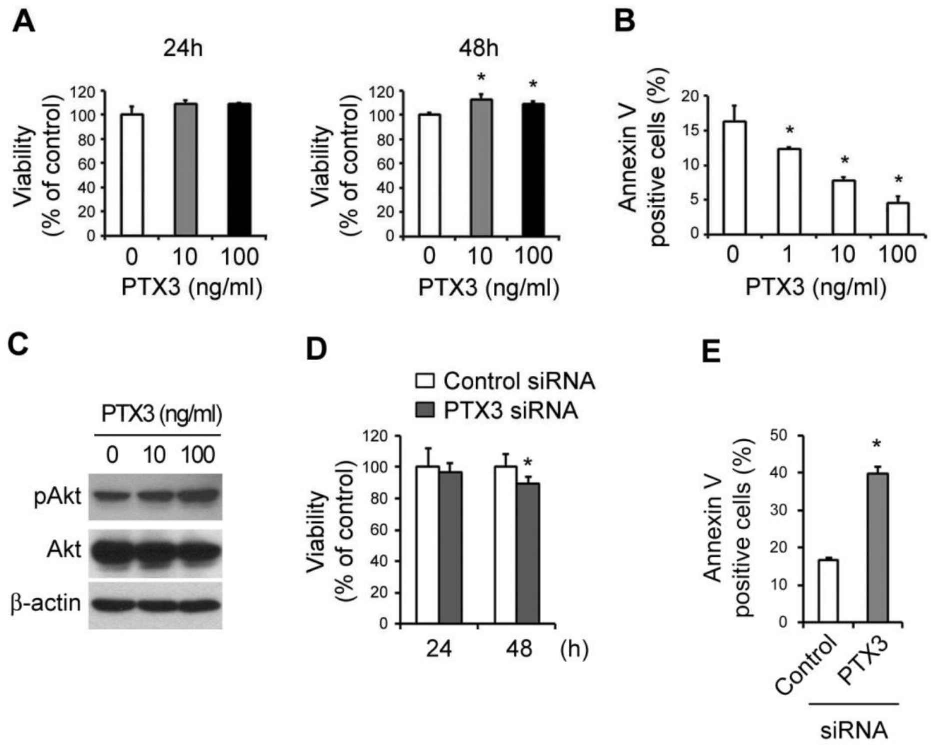

Supplementary Figure S5: Effect of PTX3 on cell proliferation and apoptosis in human gastric cancer cells. **A**. *In vitro* cell proliferation assay of HTB135 cells treated with the indicated concentrations of PTX3 for 24 and 48 hours using MTT assay. **B.** Apoptotic cell death in HTB135 cells was determined using flow cytometric analysis. HTB135 cells were treated with PTX3 (0, 1, 10, or 100 ng/ml), and apoptotic cells were detected by staining with propidium iodide (PI) and Annexin V-FITC after serum deprivation. **C**. Protein expressions of pAKT, AKT, and β-actin were detected by western blotting in HTB135 cells treated with the indicated concentrations of PTX3 for 48 hours. PTX3 induced phosphorylation of AKT in a dose-dependent manner. **D**. The effect of PTX3 silencing on proliferation of HTB135 cells was evaluated as in Figure S5A. **E**. Apoptotic cell death in HTB135 cells transfected with control or PTX3 siRNA was determined as in Figure S5B.

Supplementary Table S1: Clinical characteristics in patients with gastric cancer

Case	Age(years)	Sex	Stage	Recurrence[a]	Site of recurrence[b]	T,N,M categories[c]	Histological type
1	60	M	IB	+	Liver	T2N0M0	Intestinal
2	46	M	IB	-	-	T2N0M0	Diffuse
3	66	F	II	+	Intra-abdominal lymph node	T2N1M0	Intestinal
4	64	M	II	+	Liver, Intra-abdominal lymph node	T2N1M0	Intestinal
5	64	M	II	-	-	T2N1M0	Intestinal
6	69	M	II	-	-	T3N0M0	Intestinal
7	62	F	IIIA	+	Peritoneum	T3N1M0	Intestinal
8	72	M	IIIA	+	Peritoneum	T3N1M0	Diffuse
9	73	M	IIIB	+	Peritoneum	T3N2M0	Signet-ring cell
10	63	M	IIIB	+	Lymph node	T3N2M0	Diffuse
11	66	M	IIIA	-	-	T3N2M0	Intestinal
12	52	M	IIIA	-	-	T2N2M0	Intestinal
13	73	F	IIIA	-	-	T3N1M0	Intestinal
14	60	F	IIIB	-	-	T3N2M0	Intestinal
15	45	M	IIIB	-	-	T3N2M0	Intestinal
16	59	M	IV	+	Peritoneum	T2N3M0	Intestinal
17	59	M	IV	+	Bone, Lymph node	T3N3M0	Intestinal
18	59	M	IV	+	Bone	T3N2M0	Intestinal

[a]-: no recurrence, +: recurrence, [b]Site of recurrence after surgery, -: no metastasis, [c]Categories according to 7th edition of the AJCC cancer staging manual: stomach, T2: Tumor invades muscularis propria, T3: Tumor penetrates subserosal connective tissue, N0: No regional lymph node metastasis, N1: Metastasis in 1–2 regional lymph nodes, N2: Metastasis in 3–6 regional lymph nodes, N3: Metastasis in ≥7 regional lymph nodes, M0: Negative peritoneal cytology is classified as nonmetastatic disease.
